# Foreign–trained medical professionals: Wanted or not? A case study of Canada

**DOI:** 10.7189/jogh.03.020304

**Published:** 2013-12

**Authors:** Ruth M. Campbell–Page, Joshua Tepper, A. G. Klei, Brian Hodges, Mohammad Alsuwaidan, Dina H. Bayoumy, James A. Page, Donald C. Cole

**Affiliations:** 1 University Health Network, Toronto, Ontario, Canada; 2 Sunnybrook Health Sciences Centre, Toronto, Ontario, Canada; 3 Words Ink, Toronto, Ontario, Canada; 4 University Health Network, Toronto, Ontario, Canada; 5 Wilson Centre for Research in Education, Toronto General Hospital, Toronto, Ontario, Canada; 6 Faculty of Arts & Science, University of Toronto, Toronto, Ontario, Canada; 7 Niagara College, International Business Applied Commerce, Global Development, Niagara, Ontario, Canada; 8 Dalla Lana School of Public Health, University of Toronto, Toronto, Ontario, Canada

Definitions of the term “International Medical Graduate” vary but the Medical Council of Canada defines an IMG to be a graduate of a medical school outside of Canada or the United States, with the exception of US schools of osteopathic medicine.

There is growing global concern about the large variation among the world’s nations in the availability of physicians and the negative impact of the scarcity of physicians on health equity, health disparities, communicable and non-communicable diseases.

Many IMGs lack post–graduate residency training that is recognized as being up to Canadian standards and must therefore complete a period of remedial or post–graduate residency training before becoming certified to practice in Canada.

In Canada, the shortage of health care professionals and the dependence on international medical graduates to fill gaps in the health care work force is expected to increase in the coming years. Over the last half century there has been a trend of the migration of medical graduates from low– and middle–income countries (LMICs) to the developed world. The recipient nation and the immigrating physicians benefit from this migration, however LMICs are losing critical health capabilities as a result of the loss of these medical graduates. There is growing global concern about the large variation among the world’s nations in the availability of physicians and the negative impact of the scarcity of physicians on health equity, health disparities, communicable and non–communicable diseases. Canada, the focus of this paper, sources a significant percentage of its health care professionals from LMICs. The pathways for immigration, licensure and practice within each one of the recipient countries are complex and often not clearly laid out. As such, this paper seeks to describe a complex system – using Canada as a case study for a recipient country.

In 2005 Mullan estimated that Canada had 23.1% International Medical Graduates (IMGs) in its workforce, 43.4% of whom were immigrants from LMICs. In comparison, the United Kingdom had the highest number of IMGs in its workforce (28.3%), 75.2% of them coming from LMICs; the corresponding figures were 25.0% (60.2% from LMICs) for the United States and 26.5% (40.0% from LMICs) for Australia [1]. There are no definitive guidelines for how international health professionals are incorporated into the Canadian health system, and therefore no simple way to assess efficiency of the process or to identify best practices.

IMGs, and often their health care manager sponsors, face a complex, multi–step process to gain a Canadian medical license. Definitions of the term “International Medical Graduate” vary but the Medical Council of Canada defines an IMG to be a graduate of a medical school outside of Canada or the United States, with the exception of US schools of osteopathic medicine [2]. IMGs may be Canadian citizens or foreign citizens (**Figure 1**). The location of post–graduate training is important, including for graduates of American residency programs. Graduates of American medical school still need to complete the Royal College of Physicians and Surgeons of Canada (RCPSC) or College of Family Physicians of Canada (CFPC) certification examinations prior to qualifying for independent practice in Canada.

**Figure 1:**
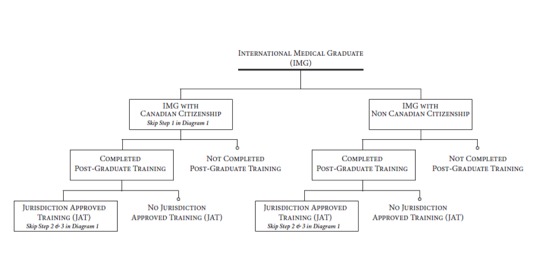
Understanding international medical graduates.

Physicians – both specialist and family – are two of 29 careers on the Citizenship and Immigration Canada’s (CIC) list of in–demand occupations for the Federal Skilled Worker Program [3,4]. The program is a gateway into Canada for individuals with language skills, education and work experience in select areas. IMGs come to Canada with various motivations, medical training regimens and areas of medical specialty. As a result, the roads for IMGs to attain a Canadian medical license are not of equal length, with each path dependent upon an individual’s unique circumstances. Occasionally, qualification to practice medicine in Canada may require an IMG to largely recomplete various parts of their training, an obstacle that can be too lengthy or costly and so may prove to be insurmountable.

IMGs generally migrate from less wealthy countries, creating a significant imbalance in the global health workforce that has long been recognized as a problem by the World Health Organization, the World Health Assembly and others [5]. Some LMIC countries have made appeals to the World Health Assembly to take measures to decrease the flow of physicians to developed countries [5]. However, the establishment of policies to respond to these appeals is a complex proposition given an individual's right to migrate to the country of their choosing. These complexities are compounded by many developed countries’ enticing recruitment strategies; images of beautiful mountain landscapes flash across the screen of *HealthMatch BC’*s website, a health professional recruitment service funded by the Government of British Columbia, Canada. Part of the mandate of *Health Match BC* is to recruit internationally educated health professionals to British Columbia [6]. Canada claims to have a similar policy – not “actively” recruiting health professionals – although as far as the primary author is concerned, this claim is dubious given the presence of external, government funded bodies whose goal is to recruit health care professionals migrate to Canada. Perhaps it depends on how one defines “active recruitment”. The primary author supposes that the Government of British Colombia as well as other provinces with these agencies do not view the use of sales techniques – beautiful landscapes and enticing descriptions of what your life will be like – as active recruitment. Which is convenient for Canada as there is increasing international concern about the large variation among the world’s nations in the availability of health care professionals, resulting in negative impact on health disparities, health equity and communicable and non–communicable diseases [2]. Some countries, such as South Africa in 1995, have taken it upon themselves to ban the recruitment of doctors from other Organization of African Unity countries [5]. The primary author encourages Canadian province and territories to clearly define and explain what their stance is on the recruitment of IMGs, as well as defining terms such as “active” if they claim not to recruit IMGs from LMICs.

## IMMIGRATION TO CANADA: THE FIRST STEP

**Figure 2:**
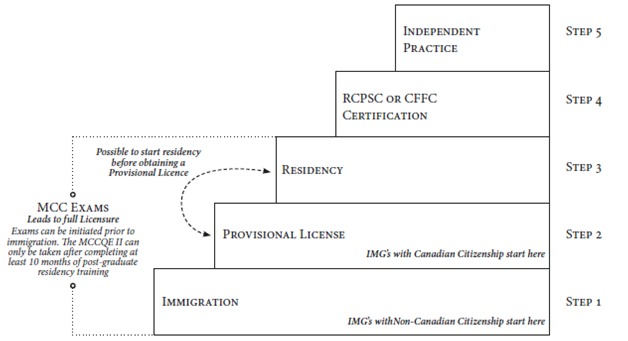
Five steps to independent practice.

IMGs who do not have Canadian citizenship must first attain an immigration status that permits them to legally remain in Canada (**Figure 2**). CIC has a number of immigration status classes. Among these, most IMGs are processed through the Economic Class (including the Federal Skilled Worker Program) and the Family Class [7]. Other routes through immigration include the Canadian Experience Class and Provincial Nominee Programs, though these are not available to all IMGs. IMGs staying with or reconnecting with family members who may be in, or moving to Canada, are eligible for permanent residency through the CIC’s Family Class, in which a relative who is a Canadian citizen or permanent resident must sponsor the applicant. Eligible relations include: common–law, same–sex and formally married spouses, dependent children, adopted children, parents and grandparents [7]. Successful applicants are given a permanent residence status. Applicants must include the results of an official language proficiency test in either French or English and have either a job offer or at least one year of continuous, paid, full–time work experience in one of the 29 in–demand occupations [7].

Instead of immediately immigrating, IMGs will often begin by obtaining a temporary work permit, which does not allow an IMG to live permanently in Canada. Still, because it can frequently be acquired more quickly than permanent residency, it is often used as a bridge towards permanent residency, allowing IMGs to begin their Canadian employment. Applications can be submitted from either outside Canada or, for those on a study visa, temporary residence or prior temporary work visa, from inside the country. In either situation applicants must have a job offer from a Canadian employer.

One route to permanent residency for individuals on a temporary work permit is the Canadian Experience Class. The Canadian Experience Class is often a gateway for temporary foreign workers or foreign students studying in Canada. It is available to skilled temporary foreign workers with at least two years of Canadian work experience in their field.

Temporary work permits are also a mechanism often used by IMGs going through a Provincial Nominee Program (PNP). Every province and territory except for Nunavut and Québec, which have different selection systems, has a PNP. The PNPs are not identical, as criteria vary between the provinces, though they do operate in a roughly similar manner. The programs are designed to help employers recruit foreign workers to occupy jobs they are having difficulty filling domestically. Finally, two other routes by which physicians could immigrate into Canada include the humanitarian class or as a refugee [8].

## OBTAINING A MEDICAL LICENSE TO PRACTICE

The Medical Council of Canada (MCC), responsible for administering national examinations and providing the Licentiate qualification for entry into practice, provides a wide range of information about the licensure process on its website. There are many steps and variables to the licensure process – that are often completed prior to arrival in Canada (**Figure 2**). Most provinces and territories have now dedicated government–funded, third party recruitment agencies to help attract IMGs to their jurisdiction. These agencies provide support and counsel through the licensure process and often host, or link to, provincial medical job boards.

In Canada, there are two categories of medical license, full and provisional. The provincial colleges of physicians and surgeons are the medical regulatory bodies and medical licensing authorities in the jurisdictions responsible for verifying credentials and determining a physician’s eligibility to practice in the provinces. An IMG must receive a license, either provisional or full, from the college before they are permitted to practice medicine. Licensure requirements are specific to each province. An IMG whose credentials meet the requirements to practice in one province, may not qualify in another [9].

A full medical license permits the physician to practice medicine with no terms, limitations or restrictions. The requirements for a full medical license are mainly uniform across Canada, though some variations do exist [10]. The main qualifications a physician must possess in order to receive a full license include: a degree in medicine from an approved medical school, completion of two years of residency training, possession of the Licentiate of the Medical Council of Canada (LMCC), and certification by examination from either the College of Family Physicians of Canada (CFPC) or the RCPSC [11]. Many IMGs need time to achieve these standards and in some jurisdictions may begin practicing in Canada on a provisional license.

A provisional license can vary from province to province; thus there are over 100 different types of provisional licenses with variables including geographic, scope of practice and location of practice restrictions. A provisional license may also be referred to as a ‘restricted’, ‘conditional’, or ‘temporary’ license [10]. A provisional license allows a physician to practice medicine, but with restrictions, including the term of the permit and geographical or other restrictions [11]. Generally, among the main differences from full licensure requirements are that the physician does not need to have received the LMCC (though they generally do need to have passed the MCC evaluating exam), completed a full two years of residency, or be certified by either the CFPC or the RCPSC and they must have an employer–sponsor. Jurisdictions vary in approach, for example in British Columbia physicians can begin practicing on a provisional license without having completed the MCC evaluating exam, provided they pass the exam within the first year, while other provinces require IMGs to have passed the first part of the MCC qualifying exam prior to granting a provisional license [12].

*National Medical Certification Exams* are one final step required before an IMG may be approved for independent practice under full licensure by a provincial or territorial regulatory authority. This is a certification via examination by one of Canada’s national certification bodies – RCPSC for specialists and CFPC for family physicians. IMGs may be permitted to practice in a province or territory without RCPSC or CFPC certification under the constraints of a provisional license [12]. IMGs interested to practice in Québec must make sure that various documentation including their medical degrees and certifications of relevant examinations has been verified by the PCRC [13].

## POST–GRADUATE RESIDENCY TRAINING

Many IMGs lack post–graduate residency training that is recognized as being up to Canadian standards and must therefore complete a period of remedial or post–graduate residency training before becoming certified to practice in Canada. The RCPSC has a jurisdiction approved training (JAT) assessment process wherein completion of post–graduate training/specialist certification in certain jurisdictions may allow an individual to bypass post–graduate training in Canada and be directly eligible for the RCPSC certification exam. These jurisdictions include: Australia, Hong Kong, Singapore, Ireland, Switzerland, the United Kingdom, New Zealand and South Africa [14]. IMGs from programs in these jurisdictions are still subject to an in–depth evaluation of their training by the RCPSC prior to having their residency training approved. On the other hand, post–graduate training in the United States is generally considered to be on par with Canadian training; therefore, IMGs from American programs may be exempt from this requirement. Regardless of citizenship, an IMG who has completed only their graduate medical training must complete post–graduate training in Canada in order to be eligible for RCPSC certification and independent licensure.

**Figure:**
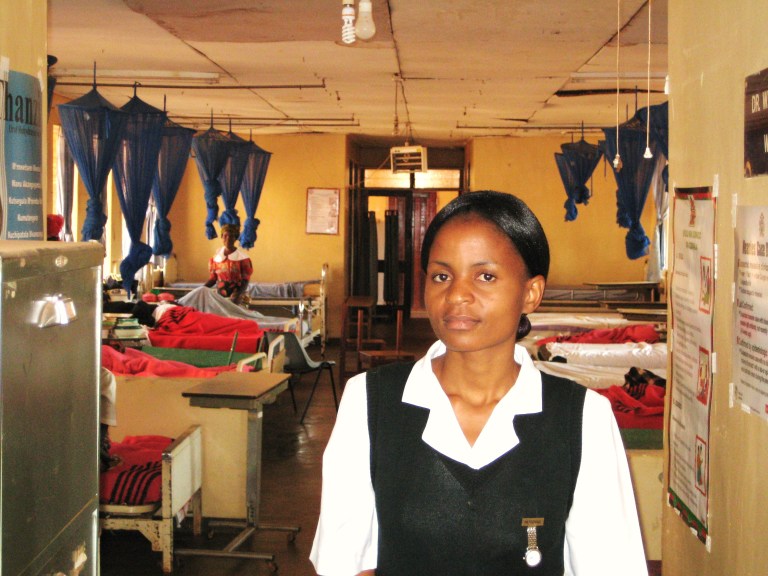
Photo: Courtesy of Alasdair Campbell, personal collection

For family physicians, the CFPC offers a program similar to the JAT called International Accreditations. Physicians with training and certification in approved jurisdictions are able to apply for certification by the CFPC without the need to complete the CFPC exam. Canada also has regulations concerning how recently a physician has practiced. If a physician has been out of practice for more than three years out of the past five they are required to redo residency training regardless of their previous training and experience. Those IMGs who do need to complete residency training will have to compete for spots through the Canadian Resident Matching Service (CaRMS). Alberta is the one exception, matching IMGs to reserved spots through its own program. To be eligible to participate in the first iteration of the CaRMS match, IMGs must have graduated from a medical school that is either accredited by the LCME (Liaison Committee on Medical Education)/CACMS (Committee on Accreditation of Canadian Medical Schools), or listed on FAIMERs International Medical Education Directory, or the World Directory of Medical Schools, and have successfully completed the MCCEE or be scheduled to write the MCCEE within the upcoming year [15].

## OBTAINING A RESIDENCY POSITION

Obtaining a residency position can be the most challenging step for many IMGs. The lack of residency positions for IMGs is consistently reported as being a major obstacle. Some provinces have a dedicated number of positions for IMGs and most provinces now allow IMGs to compete directly with Canadian medical graduates for additional placements through the CaRMS match. There may also be restrictions on residency positions based on discipline, with more spots available for family medicine than specialty disciplines. For instance, in 2010 there were 229 dedicated residency spots available for IMGs, with IMGs filling 380 total residency positions. Even so, there are vastly more IMG applicants for post–graduate medical training than there are positions. In the first iteration of the 2010 CaRMS match, 299 graduates from international medical schools, including the United States, received residency positions through CaRMS out of an applicant pool of 1532 individuals [15]. This means that for many IMGs the chances of securing a residency position without acquiring additional training or repeating medical school are slim.

## SHIFTING APPROACHES TO IMGS

In the early 1990s, a number of barriers to IMG entrance were introduced as part of a national effort to reduce the number of physicians practicing in Canada. These remained in place until the end of that decade when both the Federal government and individual jurisdictions reversed their approach to physician supply, including IMGs, and developed a broad range of measures to increase access for IMGs to the education system and the provincial/territorial licensing process [16].

Now, hundreds of internationally educated physicians go through the process of becoming certified and/or licensed to practice medicine in a Canadian province every year. From 2000–2009 Canada added 7181 IMGs to its workforce, a significant increase from the 80s and 90s when Canada gained 5216 and 4755 IMGs respectively [17]. In total, nearly one quarter of the Canadian physician workforce are IMGs [18].

Some provinces and territories use Provisionally Licensed International Medical Graduates (PLIMGs) to help meet their physician health human resource needs. Provisional medical licenses permit physicians, who may not have completed all of the certification and regulatory requirements for full, independent licensure, to practice medicine with certain restrictions.

In their article on the use of PLIMGs in Canada, Audas et al. stated in 2005: “Typically, provisionally licensed IMGs are hired to meet an immediate short fall of physicians; they obtain a provisional license to gain entry to practice and tend to fill positions that Canadian medical graduates will not take” [10,12].

More specifically, a Memorial University study, by the same researchers, showed that, “PLIMGs make up a greater proportion of the physician workforce in N.L. [Newfoundland & Labrador], compared with any other Canadian province,” [10,12] with many remaining in Newfoundland only long enough to complete their full licensing exams and then departing for more lucrative practice in other provinces as fully licensed physicians. This creates a feeder system of IMGs from some provinces to others.

A 2009 Canadian Institute for Health Information (CIHI) study on physician supply, distribution and migration corroborates this. It found that the jurisdictions with the highest percentage usage of IMGs among new physicians (Newfoundland and Labrador, Saskatchewan, Manitoba and Nova Scotia) also had the lowest ten–year retention rate of IMGs. For example, between 1995 and 1999, 60.6% of new physicians in Newfoundland and Labrador were IMGs, second to Saskatchewan with 62.2%, but ten years later the province had only retained 7 per cent of those IMGs. The study also tracked where IMGs went after leaving their first jurisdiction of registration. The results showed that, “a large proportion of new IMGs in Newfoundland and Labrador (63.3%), Nova Scotia (77.5%), Manitoba (80.4%) and Saskatchewan (92.1%) moved to Ontario, Alberta or British Columbia” [17]. Anecdotal information out of Newfoundland suggests that recently this trend is starting to change and the province is beginning to retain more of its IMGs [19].

In 2009 the Prime Minister and provincial and territorial leaders signed an update to the 1994 *Agreement on Internal Trade* (AIT) to facilitate the movement of people, investment, goods and services across Canada. The new update on labour mobility was the 9th Protocol of Amendment to the AIT and mandates that all regulated professions are entitled to full mobility rights across the country without having to undergo materially additional training, experience, examinations or assessments (barring an officially registered exception) [20]. For example, the official requirements for registration by the College of Physicians and Surgeons of Ontario (CPSO) clearly state that licensed physicians from out–of–province may apply for Ontario licensing under the provisions of the AIT. The CPSO states that, “… AIT–related provisions enable application on the basis of holding a current Canadian out–of–province license, rather than on holding the specific postgraduate Canadian qualifications that would otherwise be required” [21].

The new AIT holds the potential to greatly ease the movement of IMGs within Canada. The agreement has compelled the provinces to develop national requirements promising full mobility for health care providers, meaning that PLIMGs would have the freedom to practice medicine in the jurisdiction of their choosing. All physicians, including IMGs, would still need to complete the credentialing process and pay the required fees [21]. Provinces with greater physician needs will no longer be able to use variances in the levels of provisional licensing to attract IMGs, potentially leading to health human resource shortages in those jurisdictions. Indeed, there have been some concerns raised that a national standard for medical registration, promising unlimited mobility across Canada, could lead to an exodus of IMGs from some provinces [22].

## PROGRESS ON NATIONAL STANDARDS FOR MEDICAL REGISTRATION AND FUTURE DIRECTIONS

The mobility rights required by the AIT have prompted discussion and partnership by medical regulatory colleges across Canada, led by the Federation of Medical Regulatory Associations of Canada (FMRAC), and including the Medical Council of Canada (MCC) and Canada’s schools of medicine to create national standards for medical registration [22]. Negotiations to reach a common system have been under way for some time and progress has been made, including the development of a new, standardized application process for medical licensure now expected by early 2014 [23].

There are significant questions that have yet to be answered, including who will pay for these national standards and what will the impact be? The IT systems needed for the application process are being funded by Human Resources and Skills Development Canada, the regulatory authorities and the MCC, but no money has yet been provided to cover the anticipated costs of implementing a new nation–wide system of testing and certification [23]. What level of funding will be required and where it should come from must still be determined. Without an answer to this funding question, the date of adoption of new standards, once a common set of standards has been agreed to, is still uncertain [22].

The AIT provides a mandate for common regulation to ensure physician mobility. Agreement on a national standard for full licensure is much more easily reached than one for provisional licensure. This is partly due to the different levels of dependence on IMGs that various provinces have, to help meet their health human resource needs. As noted earlier, some provinces may receive a significant, negative impact from the granting of nation–wide mobility to provisionally licensed physicians [22].

## CONCLUSIONS

Based on all the information reviewed in this paper, it appears that Canada is continuously seeking foreign–trained medical professionals, particularly since the end of the 1990s. With an aging population leading to growing health needs this trend is set to continue. Citizenship and immigration are federally regulated so the immigration procedures described in this paper are applicable across all of Canada. By contrast, licensure varies province by province as discussed above. Information clarifying and describing the licensure process and multiple immigration entry points for IMGs will be of value for those who are in the system, for those who work with the system and for those involved in evaluating, commenting and revising the process. It must be noted that the licensure process is in flux. Furthermore, the development of a national standard for medical registration promises to greatly simplify the licensure process and level the currently uneven provincial requirements for licensure in Canada. But even once new national standards are in place, the path through immigration, residency training, licensure and employment promises to remain a difficult road to navigate.
